# Editorial: Preventing childhood asthma—the neglected impact of existing public health interventions

**DOI:** 10.3389/falgy.2025.1621332

**Published:** 2025-05-23

**Authors:** David M. Patrick, Stuart E. Turvey, Meghan B. Azad, Petra Zimmermann, Angela Dramowski, Hannah Lishman

**Affiliations:** ^1^ School of Population and Public Health, University of British Columbia, Vancouver, BC, Canada; ^2^ British Columbia Centre for Disease Control, Vancouver, BC, Canada; ^3^ Department of Pediatrics, BC Children’s Hospital, University of British Columbia, Vancouver, BC, Canada; ^4^ Manitoba Interdisciplinary Lactation Centre (MILC), Children’s Hospital Research Institute of Manitoba, Winnipeg, MB, Canada; ^5^ Department of Pediatrics and Child Health, University of Manitoba, Winnipeg, MB, Canada; ^6^ Faculty of Health Science and Medicine, University of Lucerne, Lucerne, Switzerland; ^7^ Department for Community Health, Faculty of Science and Medicine, University of Fribourg, Fribourg, Switzerland; ^8^ Department of Paediatrics, The University of Melbourne, Parkville, VIC, Australia; ^9^ Department of Paediatrics and Child Health, Stellenbosch University, Cape Town, South Africa

**Keywords:** asthma, atopic disease, antibiotics, human milk, microbiota, population

**Editorial on the Research Topic**
Preventing childhood asthma - the neglected impact of existing public health interventions

Asthma is the most prevalent chronic disease of childhood in high income countries, with prevalence rising steeply during the late 20th century, a pattern now echoed in some middle-income countries (1, 2). How can this vast burden of illness be reduced? Current strategies focus appropriately on environmental factors such as indoor and outdoor air quality, and ensuring access to effective medical treatment (3). This series summarizes growing evidence that exposure to microbes and human breastmilk during infancy shape the immune system and influence lifelong risk for atopic disease, including asthma. Such evidence begs the question: can existing public health interventions, such as reducing unnecessary antibiotic exposure during infancy and promoting breastfeeding, contribute to reversing the global pandemic of asthma and atopic disease?

Hildebrand and Adamko open the series by reviewing the changing epidemiology and distribution of asthma and allergy. The global burden of asthma and allergic disease has remained high since the late 20th century, but uneven distribution of prevalence between countries and across socioeconomic contexts highlights the complexity of early life exposures and their interaction with genetic predisposition for atopic disease.

Donald and Finlay neatly summarize crucial insight from experimental models that provide evidence of a link between antibiotic exposure, disrupted development of infant gut microbiota and subsequent risk of atopic disease. Mice without microflora have a higher risk of atopic disease, but proper immune function can be rescued by replacement of missing bacterial taxa in a critical early-life window.

Donald and Finlay’s second contribution is a detailed review of known mechanisms of immune mediation by the developing gut microbiota and its links to lifelong atopic disease risk. The human gut is the major point of contact between the immune system and the environment. Specific microbes produce metabolites, including short chain fatty acids, that “train” the immune system away from atopic responses by regulating a variety of processes, including T_reg_ development. Thus, there is a plausible pathway by which early life exposures imprint on life-long immune responses.

The global literature points to the importance of a number of pre- and perinatal exposures in predicting asthma risk, and in such studies, receipt of antibiotics during infancy has a large effect (4). Wurm et al. provide a systematic review of the effect of antibiotics on the intestinal microbiota in children. Antibiotic therapy during the first year of life clearly disrupts the gut microflora during infancy and childhood, including taxa implicated as beneficial in preventing atopic disease. Moreover, the effects on microbiota and atopic disease risk seem worse for macrolides than aminopenicillins; an important observation, because health systems are contemplating broad use of prophylactic macrolides for children in regions with possible benefit for infant mortality (5).

Brockway summarizes the potential for human milk to mitigate antibiotic-mediated atopic disease risk by preventing or repairing antibiotic-associated disruption of the infant gut microbiota. Human milk oligosaccharides, while not metabolized by the infant, favour the growth of *Bifidobacterium infantis* and other beneficial bacteria. Human milk feeding to at least six months is already recommended, but there is variation in the practice globally and limited availability of donor human milk for families unable to breastfeed.

Loutet provides a detailed study of factors associated with breastfeeding in Bangladesh, outlining the individual and system challenges in lower resource settings. Providing human milk to an infant is harder where both parents need to work, where public breastfeeding is less accepted and in the face of intensive formula marketing. In settings where antibiotic use in infancy and caesarean section rates are also high, a compounding effect on microbiome disruption must be considered when investigating the impact on atopic disease outcomes. Stronger system-level supports are required to overcome deficits in education, services and policies to promote and protect breastfeeding.

An important criticism of any theory linking antibiotics to atopic disease is the question of confounding by indication: what if the infections that caused antibiotic prescription are themselves the cause of asthma? Medeleanu et al. analyze data from a prospective birth cohort to address the interface with early-life respiratory infections and conclude that while those infections may confer independent risk for asthma, they do not explain the impact of antibiotic exposure. Further, antibiotics are associated with an array of atopic outcomes, not just asthma, speaking to the importance of a common immunomodulatory pathway.

This issue also includes articles that address additional observations about the complex pathogenesis of atopic asthma. Keleb et al. speak to the importance of outdoor environmental exposures by contributing a global systematic review and meta-analysis of the effect of pesticide exposure on childhood asthma, wheezing and respiratory infections. Jiang et al. contribute an important study on asthma severity in children with comorbid obesity in China. This reminds us that inflammation and immune modulation may be a common pathway linking asthma and obesity; both associated with disrupted microbiota.

With growing evidence of a plausible and impactful causal mechanism at play in patients and in experimental models, this issue focuses on the relevance of recent findings to population health. Dai et al. contribute important thinking on how such findings relate to health equity and cross-generational risk of chronic disease. Their article makes cogent arguments about how safeguarding the early-life microbiota may not only reduce atopic disease risk but also contribute to reducing intergenerational public health disparities.

Yang et al. contribute a regional study that characterises the burden that childhood asthma places on families through missed school days, missed work days, hospital visits and medical costs. Li et al. provide a modelling study that probes the impact of an observed 70% drop in infant antibiotic exposure on asthma burden in British Columbia, Canada. Very large drops in incident asthma cases, person-years with asthma and exacerbations have been realized, highlighting that asthma reduction should be considered in the value proposition of public health interventions such as antibiotic stewardship.

Mamun et al. address whether the observed effects of reducing exposure to antibiotics and promoting breastfeeding should have a significant effect at the population level. They observe that large declines in asthma seen in high income countries are in line with modelled predictions driven by lower exposure to antibiotics and improved uptake of breastfeeding. Falling asthma rates in British Columbia, Canada and in Germany have been linked to significant drops in antibiotic exposure (6, 7). Large drops in asthma have also been recorded in other countries where researchers are now looking for such an association (8).

Much remains to be done. Findings need to be replicated in additional cohorts and populations. Work is needed to quantify infant antibiotic exposure in resource-limited settings and identify opportunities to safely reduce unnecessary use. The complexities of competing risks are large. Antibiotic use for infants will be more important and more difficult to reduce in settings with higher sepsis rates. Approaches that have led to less antibiotic use in Canada, Germany or the UK may not be viable in other epidemiological contexts. Yet, observations outlined in this issue also remind us that atopic disease risk associated with antibiotics may be mitigated where breastfeeding or donor milk are available. A detailed understanding of beneficial bacteria and their function may also lead to better design of probiotics, prebiotics and synbiotics for the benefit of infants who need antibiotics and cannot be fed human milk.

This series provides a comprehensive introduction to experimental, population, cohort and modelling studies in this key area. When considered in aggregate, the evidence points to a significant potential to reduce the burden of atopic disease where it is possible to reduce unnecessary antibiotic use and encourage human milk feeding for infants, alongside existing evidence of the benefit of improved air quality. Antimicrobial resistance and optimal infant nutrition are already sound reasons for such actions, but it now appears increasingly likely that they may also have a sizable impact on the scale of atopic disease pandemics, morbidity and costs.

## References

[1] Platts-Mills TAE. The allergy epidemics: 1870–2010. J Allergy Clin Immunol. (2015) 136(1):3–13. 10.1016/j.jaci.2015.03.048 26145982 PMC4617537

[2] Asher MI Rutter CE Bissell K Chiang CY El Sony A Ellwood E Worldwide trends in the burden of asthma symptoms in school-aged children: global asthma network phase I cross-sectional study. Lancet. (2021) 398(10311):1569–80. 10.1016/S0140-6736(21)01450-1 34755626 PMC8573635

[3] Rodriguez A Brickley E Rodrigues L Normansell RA Barreto M Cooper PJ. Urbanisation and asthma in low-income and middle-income countries: a systematic review of the urban-rural differences in asthma prevalence. Thorax. (2019) 74(11):1020–30. 10.1136/thoraxjnl-2018-211793 31278168 PMC6860411

[4] Duong QA Pittet LF Curtis N Zimmermann P. Antibiotic exposure and adverse long-term health outcomes in children: a systematic review and meta-analysis. J Infect. (2022) 85(3):213–300. 10.1016/j.jinf.2022.01.005 35021114

[5] Keenan JD Bailey RL West SK Arzika AM Hart J Weaver J Azithromycin to reduce childhood mortality in Sub-Saharan Africa. N Engl J Med. (2018) 378(17):1583–92. 10.1056/NEJMoa1715474 29694816 PMC5849140

[6] Patrick DM Sbihi H Dai DLY Al Mamun A Rasali D Rose C Decreasing antibiotic use, the gut microbiota, and asthma incidence in children: evidence from population-based and prospective cohort studies. Lancet Respir Med. (2020) 8(11):1094–105. 10.1016/S2213-2600(20)30052-7 32220282

[7] Kohring C Akmatov MK Dammertz L Heuer J Bätzing J Holstiege J. Trends in incidence of atopic disorders in children and adolescents - analysis of German claims data. World Allergy Organ J. (2023) 16(7):100797. 10.1016/j.waojou.2023.100797 37485449 PMC10359926

[8] Kallis C Maslova E Morgan AD Sinha I Roberts G van der Valk RJP Recent trends in asthma diagnosis, preschool wheeze diagnosis and asthma exacerbations in English children and adolescents: a SABINA Jr study. Thorax. (2023) 78(12):1175–80. 10.1136/thorax-2022-219757 37524391 PMC10715559

